# Research on the Measurement Technology of Rotational Inertia of Rigid Body Based on the Principles of Monocular Vision and Torsion Pendulum

**DOI:** 10.3390/s23104787

**Published:** 2023-05-16

**Authors:** Yeqing Chen, Yi Zeng, Haoran Li, Jiye Zhang, Lieshan Zhang

**Affiliations:** School of Information Science and Engineering, Zhejiang Sci-Tech University, Hangzhou 310018, China; 2020330300298@mails.zstu.edu.cn (Y.C.); 2020330301215@mails.zstu.edu.cn (Y.Z.); 2020330300280@mails.zstu.edu.cn (H.L.); 2020330301210@mails.zstu.edu.cn (J.Z.)

**Keywords:** monocular vision, rotational inertia, torsion pendulum method, damping, rigid body, machine vision

## Abstract

Damping is an important factor contributing to errors in the measurement of rotational inertia using the torsion pendulum method. Identifying the system damping allows for minimizing the measurement errors of rotational inertia, and accurate continuous sampling of torsional vibration angular displacement is the key to realizing system damping identification. To address this issue, this paper proposes a novel method for measuring the rotational inertia of rigid bodies based on monocular vision and the torsion pendulum method. In this study, a mathematical model of torsional oscillation under a linear damping condition is established, and an analytical relationship between the damping coefficient, torsional period, and measured rotational inertia is obtained. A high-speed industrial camera is used to continuously photograph the markers on a torsion vibration motion test bench. After several data processing steps, including image preprocessing, edge detection, and feature extraction, with the aid of a geometric model of the imaging system, the angular displacement of each frame of the image corresponding to the torsion vibration motion is calculated. From the characteristic points on the angular displacement curve, the period and amplitude modulation parameters of the torsion vibration motion can be obtained, and finally the rotational inertia of the load can be derived. The experimental results demonstrate that the proposed method and system described in this paper can achieve accurate measurements of the rotational inertia of objects. Within the range of 0–100 × 10^−3^ kg·m^2^, the standard deviation of the measurements is better than 0.90 × 10^−4^ kg·m^2^, and the absolute value of the measurement error is less than 2.00 × 10^−4^ kg·m^2^. Compared to conventional torsion pendulum methods, the proposed method effectively identifies damping using machine vision, thereby significantly reducing measurement errors caused by damping. The system has a simple structure, low cost, and promising prospects for practical applications.

## 1. Introduction

Rotational inertia is a physical quantity that characterizes the magnitude of an object’s inertia during rotational motion around an axis. It is a measure of the rotational performance of self-propelled equipment. Like mass, center of mass, and product of inertia, it is a mass characteristic parameter of objects. Rotational inertia is an inherent property of any object with mass [1,2,3,4]. The role of rotational inertia in rotational motion is analogous to the role of mass in linear motion. It is an essential parameter in the dynamic modeling and analysis of rotating rigid bodies [5,6,7]. For example, rotational inertia is crucial in various applications, such as gyroscopes [8], celestial bodies [9], and motor rotors [10]. Furthermore, a rotational inertia test is required for all equipment with rotational behavior, such as spacecrafts [11], aircrafts [12], automobiles [13], robots [14], specialized helmets [15], and tennis rackets [16].

The rotational inertia of an object is related to its mass, the position of its rotation axis, and the distribution of its mass. For rigid bodies with complex shapes and nonuniform mass distributions, experimental methods are usually required to determine the rotational inertia [17]. In an experiment, the object under test is generally set in motion in a certain way, and the measurement of rotational inertia is obtained from the mathematical relationship between motion characteristics and rotational inertia. The torsion vibration response method is a commonly used mechanical performance testing method in engineering [18], and different torsion vibration testing methods, including the multiple pendulum method [13], compound pendulum method [19], and torsion pendulum method [20], are often used for testing inertial parameters. Among them, the torsion pendulum method is currently the most accurate and reliable method for measuring rotational inertia and is widely used in the measurement of the rotational inertia of large-sized equipment [21,22,23]. Regardless of which torsion vibration testing method is used, the calculation of rotational inertia is based on the relationship between measured rotational inertia and the torsion vibration motion period.

In recent years, scholars have focused on three fields of research on the measurement technology of rotational inertia: The first field is integrated test technology for measuring the mass characteristic parameters of large-scale objects. For example, Zhang et al. [21] designed and studied an integrated measurement system for the measurement of the mass characteristic parameters of high-mass nonrotary aircrafts, which can complete the measurement of mass, center of mass, rotational inertia, and product of inertia in a single hoisting cycle. Teng et al. [24] combined the multi-point weighing method and torsion pendulum method to realize the integrated measurement of satellite mass characteristic parameters. Olmedo et al. [25] studied an experimental test method for the mass characteristic parameters of robots and developed a set of torsion pendulum test platforms. The second field is online or on-orbit identification technology for measuring inertial parameters. For example, Jin et al. [26] proposed a method based on a double unscented Kalman filter (DUKF) for the online identification of lightweight electric vehicle inertial parameters to solve the impact of a sharp reduction in vehicle mass and body size on the identification of the inertial parameters during operation. Manshadi et al. [27] studied a nonlinear filtering method for estimating aircraft mass properties during airdrop maneuvers. In the first step of the method, a single extended Kalman filter is used to estimate the total mass and moment of inertia of an aircraft before the start of an airdrop maneuver. In the second step, a joint extended Kalman filter is employed to estimate the dynamic state and mass parameters of the aircraft during the airdrop maneuver. The third field is research on the damping effect in the measurement process of the torsion pendulum method and corresponding error compensation techniques. For example, Gandino et al. [28] studied the structural damping effect in a time-varying inertia complex pendulum torsion vibration system, gave an analytical model of the torsion vibration system, and defined the equivalent damping ratio from the energy point of view. Zhao et al. [29] studied the nonlinear damping effect in moment of inertia measurement by the torsion pendulum method and proposed a measurement error compensation model. With the gradual application of air bearings to torsion pendulum test benches, the frictional damping of torsion vibration has been so reduced that it can be ignored. However, for objects with complex aerodynamic shapes, air damping during torsion vibration will also affect the measurement of rotational inertia. In order to minimize the influence of damping on measurement, sensors must be used to accurately record the torsion pendulum curve. Vibration damping is estimated by the attenuation of the angular displacement amplitude, so as to eliminate or compensate for measurement error. In engineering, grating displacement sensors or angle encoders are often used to accurately record the angular displacement curve of torsion vibration [23,30]. The use of these sensors greatly increases both the cost of the measurement system and the complexity of the measurement algorithms.

Vision measurement technology utilizes high-precision industrial cameras to capture images of a measured object and obtains the dimensional parameters of the object through the object–image relationship of the imaging system. When combined with artificial intelligence algorithms, it forms machine vision technology, which plays an irreplaceable role in an increasing number of dimensional measurement applications [31,32,33,34]. In this paper, monocular vision technology is introduced into the measurement of rigid bodies’ rotational inertia and is used to realize the real-time recording of the torsion vibration angle displacement and to obtain the torsion pendulum curve. This approach can not only accurately determine the torsion vibration period of an object but also map the changes in relevant parameters of the object under the influence of damping, thus achieving an accurate measurement of rotational inertia.

## 2. Principle of the Torsion Pendulum Method for Measuring Rotational Inertia

**Figure 1:**
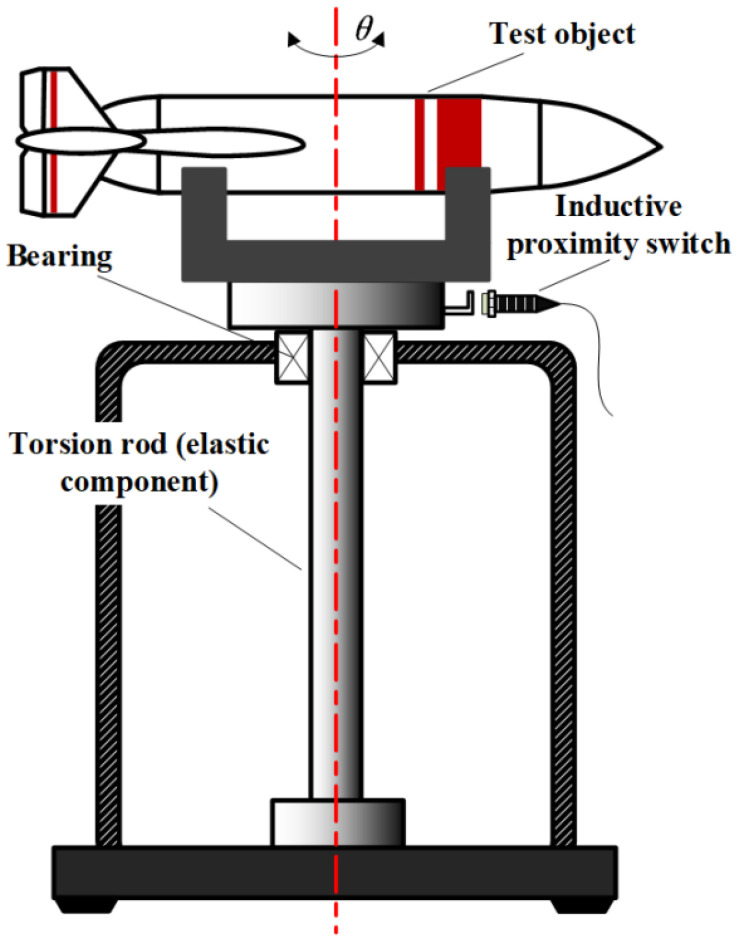
Schematic diagram of rotational inertial measurement using torsion pendulum method.

As shown in Figure 1, the principle of measuring rotational inertia by the torsion pendulum method is described using a torsion pendulum measuring table as an example. The core component of the torsion pendulum measuring table is an elastic element (usually a torsion bar or torsion spring). When the load platform and the measured object rotate around the axis of the torsion pendulum by a certain angle *θ*, the elastic element continuously converts kinetic energy into potential energy, driving the measured object into reciprocating torsion vibration. When the damping effect is ignored, the measured rotational inertia is proportional to the square of the torsion vibration period. The torsion vibration period is often counted using a proximity switch as shown in the figure below.

The torsion vibration system is a typical single-degree-of-freedom system, and the torsion angle *θ* can be defined as its generalized coordinate. Therefore, according to the Lagrangian equation, we have the following:

(1) $$\frac{d}{dt}\left(\frac{\partial L}{\partial \dot{\theta }}\right)-\frac{\partial L}{\partial \theta }=Q,$$

where *L* represents the Lagrangian function. *L* = *T* − *V*, where *T* is the kinetic energy of the system, *V* is the potential energy of the system, *Q* is the generalized external force, and *t* is time. In the torsion vibration system, the kinetic energy *T* can be expressed as

(2) $$T=\frac{1}{2}I{\dot{\theta }}^{2},$$

where *I* is the rotational inertia of the load. Neglecting the nonlinear damping of the torsion vibration system, we have the following:

(3) $$V=\frac{1}{2}k\theta^{2},$$

where *k* is the stiffness coefficient of the elastic element, which is related to the mechanical properties of the material and the length and diameter of the elastic element. The generalized force of the torsion vibration system is the damping torque, which is composed of air damping caused by the shape of the object being tested, bearing friction damping, and the internal damping of the elastic element. It often takes the form of linear damping proportional to the torsion vibration angular velocity. It can be expressed as

(4) $$Q=-c\dot{\theta }.$$

By combining Equations (1)–(4), the differential equation of torsion vibration motion can be obtained as follows:

(5) $$I\ddot{\theta }+c\dot{\theta }-k\theta =0.$$

For the torsion vibration system, assuming that the initial deflection angle is $\theta \left(0\right)=\theta_{0}$ and the initial angular velocity is ${\left.\frac{d\theta }{dt}\right|}_{t=0}=0$, the solution to the differential equation can be obtained as follows:

(6) $$\theta \left(t\right)=\theta_{0}\exp\left(-\frac{c}{2I}t\right)\cos\left[\sqrt{\frac{k}{I}-{\left(\frac{c}{2I}\right)}^{2}t}\right].$$

As shown in Equation (6), when the damping factor is considered, the curve of torsion pendulum angle displacement over time is a modulated asymptotic single-frequency curve with a frequency of $\sqrt{\frac{k}{I}-{\left(\frac{c}{2I}\right)}^{2}}$ and an initial phase of 0. Using displacement sensors, such as grating sensors, it is easy to sense the torsion pendulum angle displacement. When the damping is small, the torsion pendulum angle displacement signal is a narrowband signal, and its instantaneous characteristics can be identified using the Hilbert transform. Thus, it is possible to estimate the parameters of objects with time-varying inertial parameters.

Let *ω_m_* be the main frequency of the torsion pendulum and *ζ* be the amplitude modulation parameter that reflects the effect of damping. Let *t_n_* be the time corresponding to the nth maximum (or minimum) value of the angular displacement curve, and let *θ_n_* be the corresponding angular displacement. Then, we have the following:

(7) $$\left\{\begin{array}{c} \omega_{m}=\frac{2\pi n}{t_{n}}\\ \zeta =\frac{\ln\left(\frac{\theta_{n}}{\theta_{0}}\right)}{t_{n}} \end{array}\right..$$

Therefore, by extracting the extremum points of the torsion pendulum angular displacement curve, it is possible to calculate the main frequency *ω_m_* and amplitude modulation parameter *ζ* of the torsion pendulum and thus determine the rotational inertia *I* of the tested load. The calculation formula is as follows:

(8) $$I=\frac{k}{\omega_{m}^{2}+\zeta^{2}}.$$

As a result, the torsion pendulum frequency *ω_m_* can also be measured by extracting the zero-crossing points of the torsion pendulum angle displacement curve. Therefore, accurately recording the angle displacement curve of the torsion pendulum is the key to measuring the rotational inertia.

## 3. Principle of Recording Torsion Pendulum Curve Based on Monocular Vision

**Figure 2:**
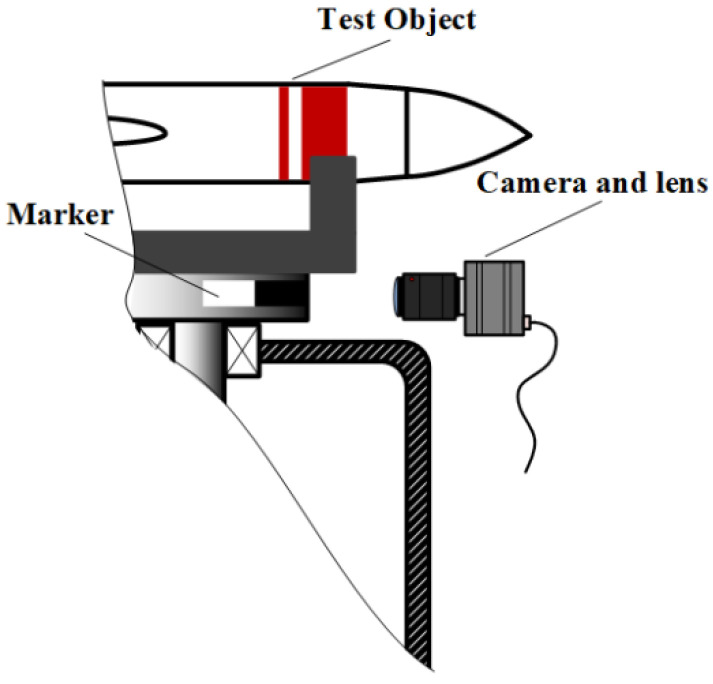
Schematic diagram of recording torsion pendulum angular displacement.

Monocular vision measurement technology can detect and record the size information of a measured object. This paper applies it to the recording of the torsion pendulum angle displacement curve. As shown in Figure 2, when monocular vision is used to record the torsion pendulum curve a marker is first set at the edge of the load table of the torsion pendulum measurement platform. Then, the marker is continuously captured using a high-speed camera and an imaging lens.

### 3.1. Image and Coordinate System Conversion Relationship

**Figure 3:**
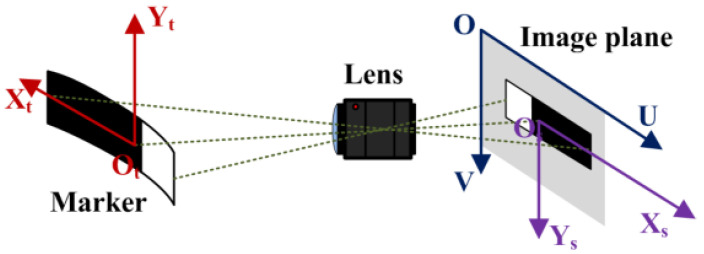
Schematic diagram of the object coordinate system and the image coordinate system.

The monocular vision measurement technology is theoretically built on a pinhole imaging model. To accurately describe the relationship between the object and image, it is necessary to establish an accurate transformation relationship between the object plane coordinate system and the pixel coordinate system. The definitions of the relevant coordinate systems are shown in Figure 3.

O-UV in the figure is called the pixel coordinate system, which reflects the arrangement of pixels in the camera’s CCD/CMOS chip. Its origin is located at the upper-left corner of the image, and the U and V axes are parallel to the two sides of the image plane, with coordinate values as integers representing pixel numbers. O_s_-X_s_Y_s_ is called the image plane coordinate system, which is also a two-dimensional Cartesian coordinate system with two axes parallel to the U and V axes of the pixel coordinate system, respectively. Its origin is located at the intersection of the optical axis and the image plane of the imaging system. O_t_-X_t_Y_t_ is called the object plane coordinate system. For convenience, its two axes are set parallel to the two axes of the image plane coordinate system, respectively, and its origin is located at the intersection of the optical axis and the marker.

According to the principle of pinhole imaging, the coordinates (*x_t_*,*y_t_*) of a point in the object plane can be transformed into the coordinates (*u*,*v*) in the pixel coordinate system of the image, as shown in Equation (9):

(9) $$U_{0}\left[\begin{array}{c} u\\ v \end{array}\right]=\left[\begin{array}{cccc} f_{x} & 0 & u_{0} & U_{0}u_{0}\\ 0 & f_{y} & v_{0} & U_{0}v_{0}\\ 0 & 0 & 1 & U_{0} \end{array}\right]\left[\begin{array}{c} x_{t}\\ y_{t}\\ 0\\ 1 \end{array}\right],$$

namely,

(10) $$\left\{\begin{array}{c} x_{t}=\frac{U_{0}\left(u-u_{0}\right)}{f_{x}}\\ y_{t}=\frac{U_{0}\left(v-v_{0}\right)}{f_{y}} \end{array}\right..$$

In the equation, *U*_0_ represents the object distance, and *u*_0_,*v*_0_ represents the coordinate position of the origin of the image plane coordinate system in the pixel coordinate system. These parameters can be calibrated using Zhang’s calibration method [35]. *f_x_* is the normalized focal length in the U-axis direction, *f_x_* = *f*/d*x*, *f_y_* is the normalized focal length in the V-axis direction, with *f_y_* = *f*/d*y*, where *f* is the focal length of the lens, and d*x* and d*y* are the sizes of the image pixels in the two directions. Equation (10) establishes a one-to-one correspondence between the pixels in the image and the points on the object plane.

### 3.2. Method for Calculating the Angular Displacement of Torsion Motion

In this paper, black and white pattern boundaries are used as the marker to record torsion vibration movements. When the camera system is set, the marker is adjusted to align perfectly with the vertical axis of the image plane coordinate system. The center point of the marker is used as the measurement point to calculate the torsion angular displacement. During the torsion pendulum, only the changes of the horizontal coordinate component of this point need to be considered.

**Figure 4:**
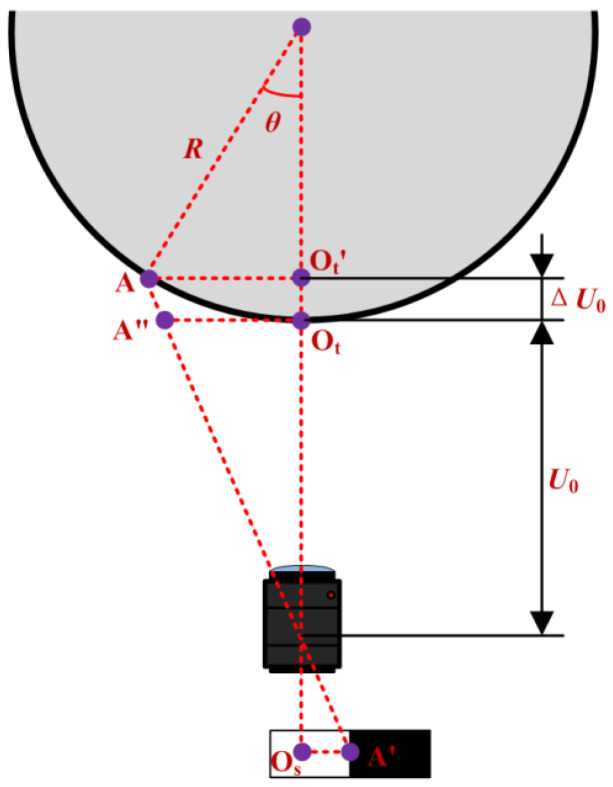
Schematic diagram of the calculation of torsion angular displacement.

Since the marker is pasted on a cylindrical surface, the center point of the marker deviates from the initial zero position when torsion pendulum occurs, resulting in a change in the object distance of the imaging system. As shown in Figure 4, it is assumed that the center point of the marker is A, its coordinates in the object plane coordinate system are *x*_1_,*y*_1_, and the corresponding image point is A’. The pixel coordinates of A’ are *u*_1_,*v*_1_, and the coordinates of A” can be obtained according to Equation (10).

Based on the geometric relationship shown in Figure 4, the formula for calculating the torsion angular displacement *θ* is as follows:

(11) $$\theta =arctan\frac{x_{1}-x_{0}}{R},$$

where *x*_0_ represents the horizontal coordinate of the center point of the marker at the initial position. The polarity of the angular displacement is determined by the polarity of the X-axis coordinate value of point A, and the initial torsion angle is defined as the positive polarity direction.

As shown in the figure, the change in object distance Δ*U*_0_ can be calculated using the following equation:

(12) $$\Delta U_{0}=\left(1-\cos\theta \right)R.$$

Further derivation can be obtained as follows:

(13) $$R\tan\theta =\frac{\left[U_{0}+\left(1-\cos\theta \right)R\right]\left(u_{1}-u_{0}\right)}{f_{x}}-x_{0}.$$

As seen from Equation (13), there is only one unknown variable *θ*, and the angular displacement at each time point can be accurately calculated by using the table lookup method.

In engineering practice, to ensure safety during measurement of the moment of inertia of large-mass objects, the pendulum angle is generally limited to a range of ±5°, and cosθ corresponding to this range is approximately equal to 1, so the angular displacement can be approximately calculated using Equation (14):

(14) $$\theta =arctan\frac{U_{0}\left(u_{1}-u_{0}^{\prime }\right)}{Rf_{x}},$$

where ${u^{\prime }}_{0}$ represents the horizontal pixel coordinate of the center of the marker at the initial position, and this value is 0 when the initial position is ideally aligned.

### 3.3. Image Processing Algorithm

**Figure 5:**
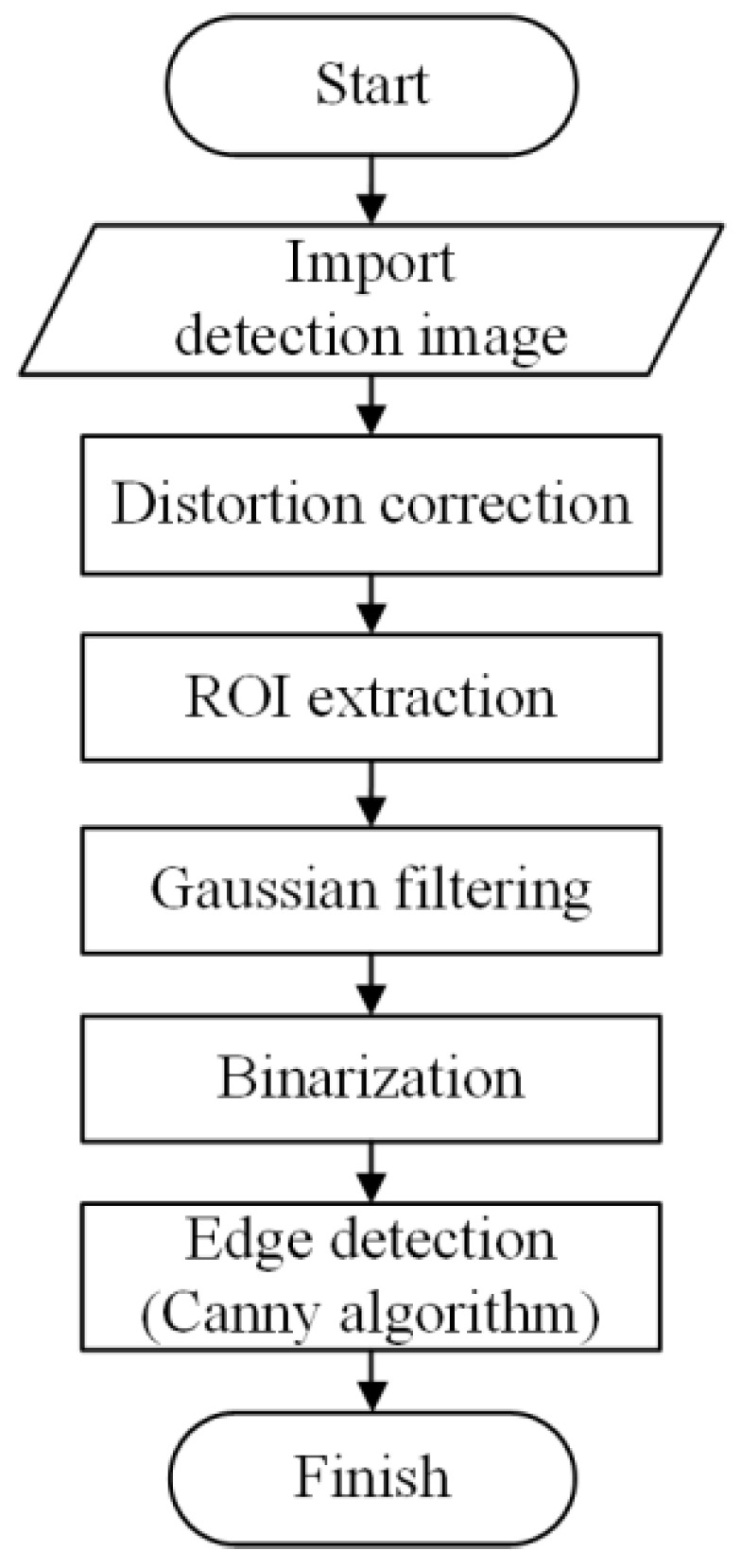
Flowchart of image preprocessing.

The main purpose of the image processing algorithm is to extract the center point of the marker in each frame of image. In order to make the marker features more distinct, the preprocessing of the digital image of the collected points is required, as shown in Figure 5. This mainly includes image distortion correction, ROI extraction, Gaussian filtering, binarization, and Canny edge detection.

Image correction refers to the correction calculation of the image based on distortion coefficients calibrated by the camera. The purpose of Gaussian filtering is to convolve the original image matrix with a weight matrix based on Gaussian distribution, which helps reduce noise generated by the camera and environment. Image binarization sets the grayscale of different pixels in the image to either 0 or 255 based on calculation with a certain threshold, which highlights the features of the measured marker. Morphological operations are simple operations based on the shape of the image, such as dilation, erosion, and opening and closing operations. The Canny edge detection algorithm is used to extract the contour of the measured marker.

**Figure 6:**
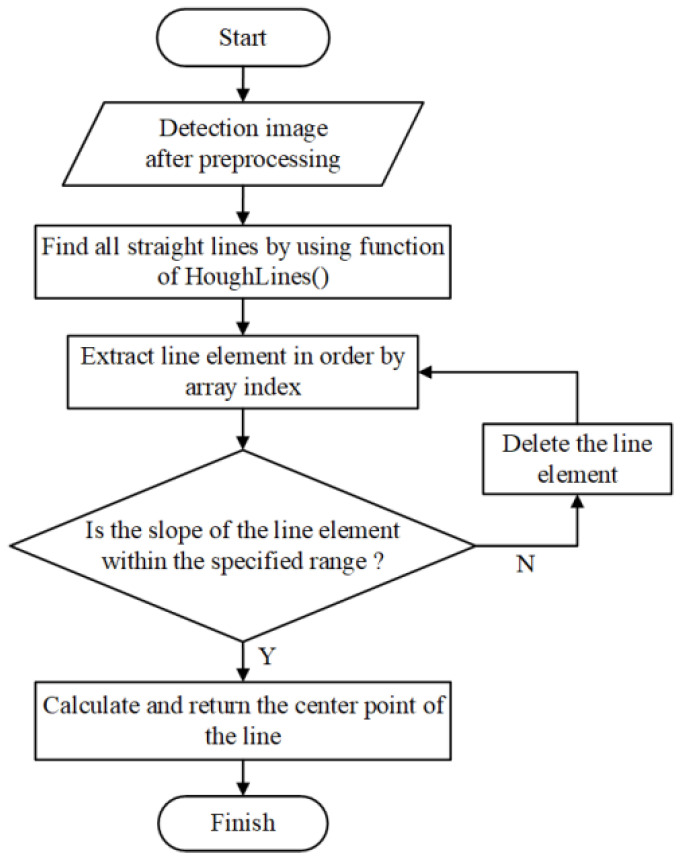
Flowchart of marker center extraction processing.

In this paper, the powerful image processing capabilities of the OpenCV library are utilized to further treat the preprocessed images. The algorithm for extracting the coordinates of the center point of the marker is shown in Figure 6. This paper mainly uses the HoughLines() function of the Hough transform to find the straight lines in the preprocessed image and determines whether they are marker straight lines based on the slope of the lines. If a line is a marker straight line, the center point of the line is returned as the coordinates of the marker center.

Based on the analysis presented earlier, the overall algorithm of the proposed method for measuring rotational inertia based on monocular vision and torsion pendulum principles can be obtained. The measurement algorithm is briefly described as follows:

Obtain the detection video and extract a frame of the image at a pertinent time point. Preprocess the image and call the algorithm for extracting the center point of the marker. Calculate the torsion angular displacement under the current image condition based on Formula (14). Repeat the above steps to complete the processing of all images, obtain the sequence of torsion angular displacement and time *θ*(*t*), draw a torsion pendulum curve, extract the zero and pole points of the torsion motion angular displacement curve, and calculate the measured rotational inertia based on the formula for calculating rotational inertia.

## 4. Experimental Verification

### 4.1. Experiment System

**Figure 7:**
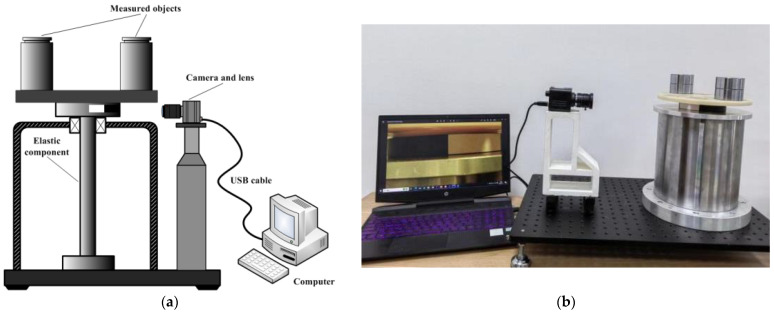
(**a**) Schematic diagram of the experimental system; (**b**) physical diagram of the experimental system.

To verify the accuracy of the method proposed in this paper, an experimental system was built as shown in Figure 7. The system mainly consists of three parts: (1) a torsion pendulum measurement platform with an elastic element as its core, which uses a large torque torsion spring as the elastic element; (2) a torsion motion recording and imaging system consisting of a high-speed industrial camera and an imaging lens, with the main parameters of the imaging system shown in Table 1; (3) a computer and software system used to process the measured images and calculate the rotational inertia of the measured object.

After calibration, the optical magnification β of the constructed imaging system is about 0.1, and the size resolution of the object plane is about 0.035 mm. According to the radius of the cylinder that the marker is pasted on, the resolution of angular displacement measurement can be calculated to be about 0.02°. In addition, more angular resolutions can be obtained by adjusting the distance between the camera and the detected object. Based on the camera frame rate, the time interval between adjacent detected frames is about 0.0167 s. After algorithm refinement, the measurement resolution of the torsion period is about 0.008 s, which can fully meet the requirements for measuring the main frequency of torsion motion. Although the sampling time interval of the camera is not absolutely uniform, the period calculation of torsion vibration is taken from multiple complete sinusoidal waveforms, so the inertia measurement error caused by uneven samplings can be ignored. To measure various rotational inertias, standard specimens with regular shapes (cylinders) and uniform mass distributions are prepared. The relative true values of the measured rotational inertia in the following experiments are all obtained by theoretical calculations.

### 4.2. Calibration of the Stiffness Coefficient and the No-Load Rotational Inertia of the Elastic Element

Based on Equation (8), further derivation can be obtained as follows:

(15) $$k=I\left(\omega^{2}+\zeta^{2}\right).$$

The torsion dominant frequency and modulation parameter *ζ* in the equation can be obtained by analyzing the torsion angle displacement curve. In order to obtain the stiffness coefficient *k*, only one known measured moment of inertia is needed.

Suppose the unloaded moment of inertia of the measuring table is *I*_0_, the torsion natural frequency is *ω_m_*_0_, and the modulation parameter is *ζ*_0_ under an unloaded condition. If the measured moment of inertia is *I*_1_, the calculated torsion main frequency will be *ω_m_*_1_, and the modulation parameter will be *ζ*_1_. The unloaded moment of inertia and the stiffness coefficient of the elastic element can be calculated by Equation (16):

(16) $$\left\{\begin{array}{c} I_{0}=\frac{I_{1}\left(\omega_{m1}^{2}+\zeta_{1}^{2}\right)}{\left(\omega_{m0}^{2}+\zeta_{0}^{2}\right)-\left(\omega_{m1}^{2}+\zeta_{1}^{2}\right)}\\ k=\frac{I_{1}\left(\omega_{m1}^{2}+\zeta_{1}^{2}\right)\left(\omega_{m0}^{2}+\zeta_{0}^{2}\right)}{\left(\omega_{m0}^{2}+\zeta_{0}^{2}\right)-\left(\omega_{m1}^{2}+\zeta_{1}^{2}\right)} \end{array}\right..$$

**Figure 8:**
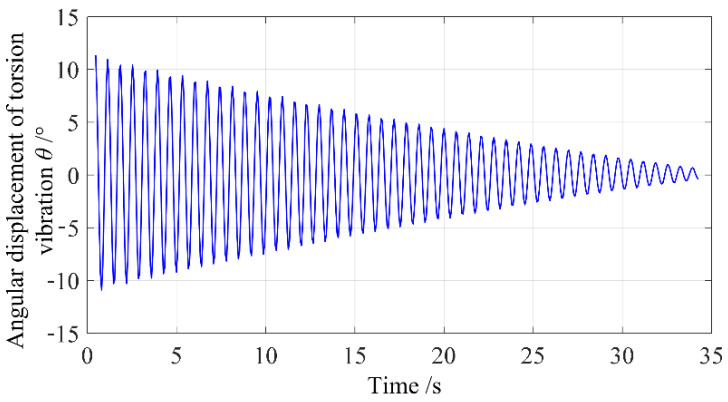
Measured torsion pendulum motion curve in no-load state.

In this paper, a pair of uniformly dense and standard cylindrical metal bodies are used to calibrate the unloaded moment of inertia and stiffness coefficient of the elastic element. The basic parameters of these two standard specimens are as follows: The mass of the standard specimens are 0.5021 kg and 0.5006 kg, their diameters are 60.07 mm and 60.04 mm, and their moments of inertia about their own rotational axes are 2.265 × 10*^−^*^4^ kg·m^2^ and 2.256 × 10*^−^*^4^ kg·m^2^, respectively. To keep the bias within a controllable range, the two measured standard specimens are symmetrically placed on the load platform, and the distance between their center axes and the center of the torsion pendulum axis is denoted as L. The measurement system can obtain different values of the loaded moment of inertia when the center distance varies. Stiffness coefficient calibration experiments were performed in an unloaded state and three different loaded states, and the calibration data of the stiffness coefficient are shown in Table 2. The torsion oscillation motion captured by the imaging system in the unloaded measurement is shown in Figure 8. The relative true values of the measured moment of inertia in the table are calculated based on the parallel axis theorem. The averages of the three calibration results are taken as the calibration values of the practical unloaded moment of inertia and stiffness coefficient of the elastic element. As shown in Figure 8, the machine vision method described in this paper can capture the torsion angle displacement very well, and the characteristics of the measured torsion angle displacement curve are completely consistent with the theoretical analysis.

### 4.3. Correction of the Pose Deviation of the Imaging System

**Figure 9:**
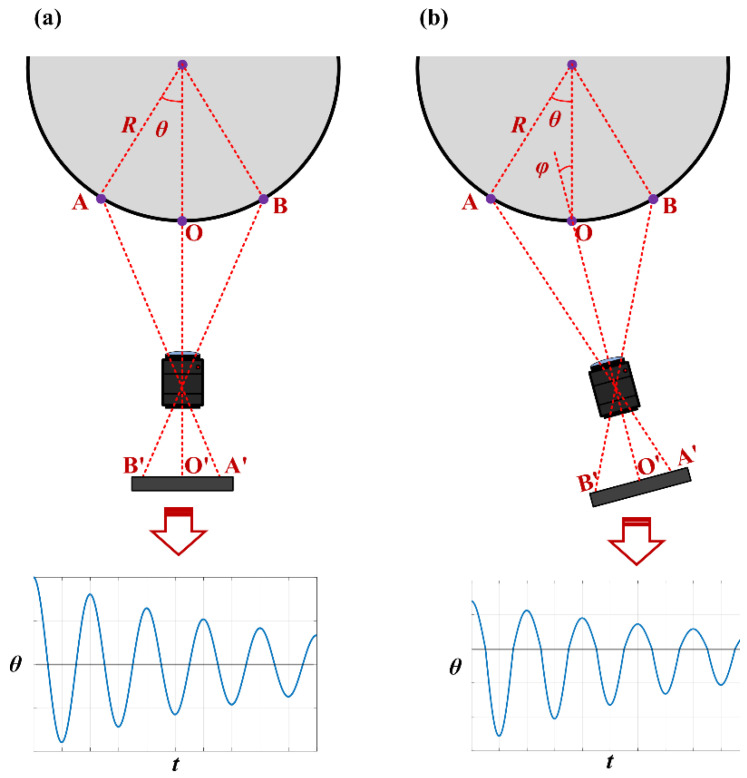
(**a**) The imaging system is in the normal pose; (**b**) the imaging system has a pose error.

The method described in this paper has certain requirements for the pose of the imaging system. As shown in Figure 4, at the initial position it is necessary to ensure that the optical center of the imaging lens, the center point of the marker, and the center of the torsion pendulum are located on the same straight line, and the optical axis of the imaging system is required to be parallel to the line connecting the center point of the marker and the center of the torsion pendulum. In the experiment, before the measurement it is necessary to adjust the pose of the imaging system by turning on the camera to observe the position of the captured marker in the image and adjusting the pose of the imaging system so that the centerline of the marker is exactly in the middle of the image and parallel to the U-axis of the image. After such adjustment, there may still exist an angle deviation of the optical axis of the imaging system relative to the ideal position (as shown in Figure 9a), which is the pose error (as shown in Figure 9b).

Figure 9 depicts the torsion swing displacement curves that can be measured by the imaging system in the ideal state and with a pose error, respectively. Comparing these two cases shows that the main frequency of the torsion vibration does not change when the imaging system has a pose deviation, but significant errors occur in the calculation of the amplitude modulation parameter. For the case of large loads, such errors in the amplitude modulation parameter are intolerable, and it is necessary to adjust the pose of the imaging system.

**Figure 10:**
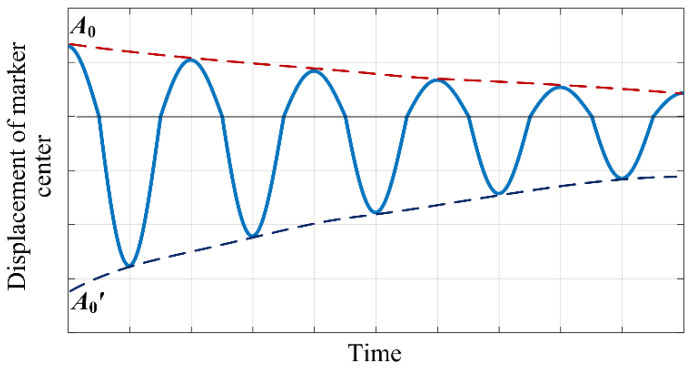
Diagram of the displacement curve of the marker center point when the imaging system has pose errors.

Figure 10 illustrates the torsion swing displacement curve when the imaging system’s optical axis has a counterclockwise deviation angle relative to the line connecting the center of the marker and the center of the torsion pendulum. It can be seen that when there is a pose error in the imaging system the amplitude and decay rate of the torsion swing displacement in the positive and negative directions are different, and the attenuation coefficients of the upper and lower envelope curves of the torsion swing displacement curve are also different. In order to estimate the deviation angle of the imaging system’s optical axis, it is necessary to obtain the amplitude of the torsion swing displacement in the clockwise and counterclockwise directions (i.e., the positive and negative polarities of the angular displacement) under a zero-damping condition. The amplitude of the torsion swing displacement in the clockwise direction is the initial displacement of the excitation position, as indicated by *A*_0_ in Figure 10; when the damping effect is significant, the initial amplitude in the counterclockwise direction can be calculated by the lower envelope curve, as indicated by *A*_0_’ in Figure 10.

The mathematical model for the lower envelope of the torsion pendulum curve is shown in Equation (17):

(17) $$y=A_{0}^{\prime }exp\left(-\xi t\right),$$

where *ξ* represents the attenuation coefficient. By capturing the torsion pendulum displacement curve using the imaging system, extracting the minimum points, and substituting two minimum points with the maximum time interval into Equation (17), the initial amplitude *A*_0_’ and attenuation coefficient *ξ* can be calculated. After obtaining the initial amplitude in clockwise and counterclockwise directions, the deflection angle of the imaging system’s optical axis can be calculated according to the following equation:

(18) $$\phi =\frac{arctan\frac{A_{0}}{U_{0}}-arctan\frac{\left|A_{0}^{\prime }\right|}{U_{0}}}{2}.$$

The sign of the angle *ϕ* indicates the direction of the deviation of the imaging system’s optical axis relative to the line connecting the center of the marker and the center of the torsion pendulum. A positive angle represents clockwise deviation, while a negative angle represents counterclockwise deviation. Based on the estimated deviation angle, the pose of the imaging system can be corrected.

### 4.4. Rotational Inertia Measurement Experiments

**Figure 11:**
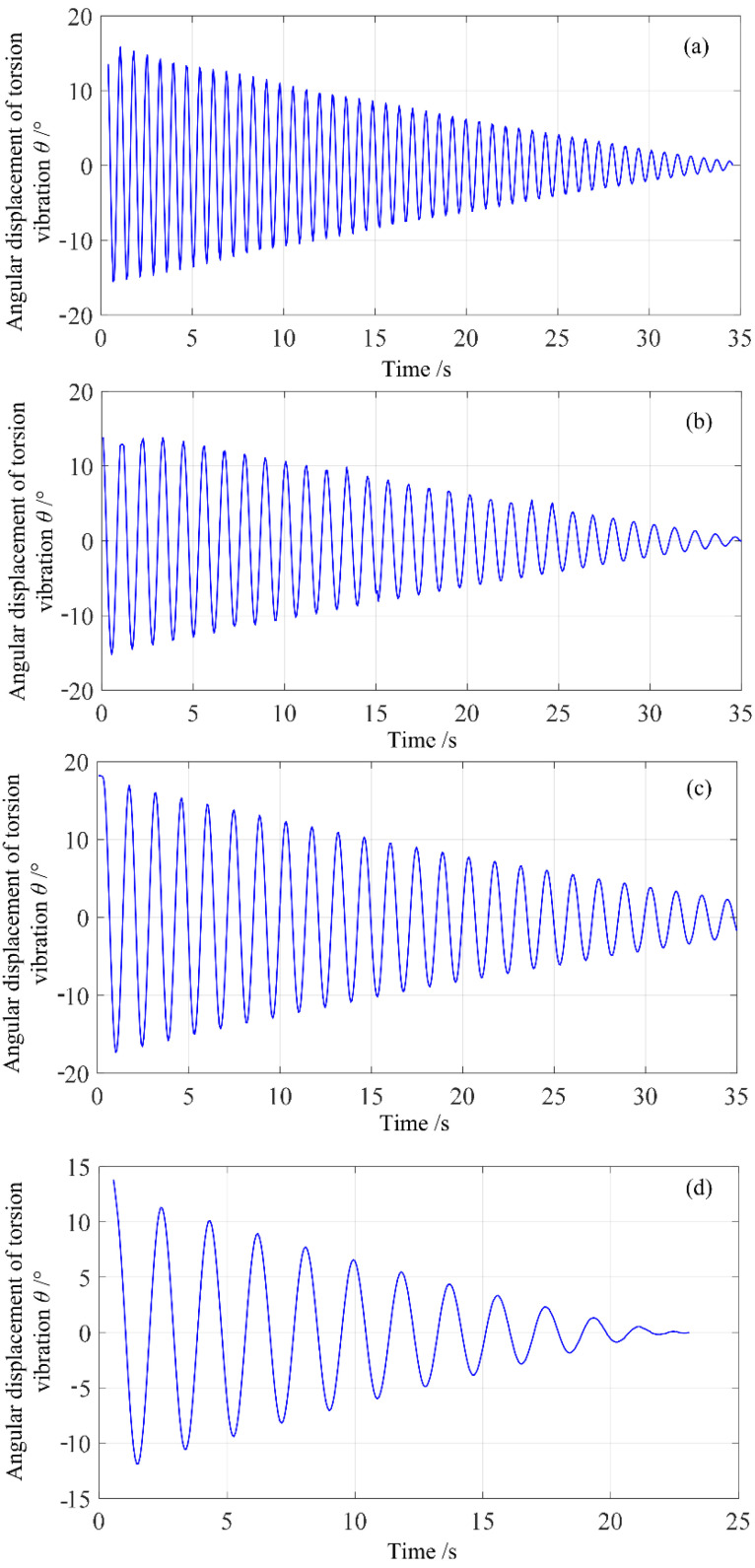
Measured angle displacement curves of torsion pendulum in 4 different load states.

To further verify the effectiveness of the proposed method, multiple measurement experiments were carried out under different load conditions. Figure 11 shows the torsion pendulum angular displacement curves captured by the imaging system under four different load conditions. The relative true values of the measured moment of inertia for these four load conditions are as follows: (a) 4.519 × 10*^−^*^4^ kg·m^2^; (b) 6.359 × 10*^−^*^4^ kg·m^2^; (c) 14.452 × 10*^−^*^4^ kg·m^2^; (d) 28.238 × 10*^−^*^4^ kg·m^2^. It can be observed from the figure that as the measured moment of inertia increases, the main frequency of torsion pendulum gradually decreases, and the amplitude attenuation becomes more significant.

**Figure 12:**
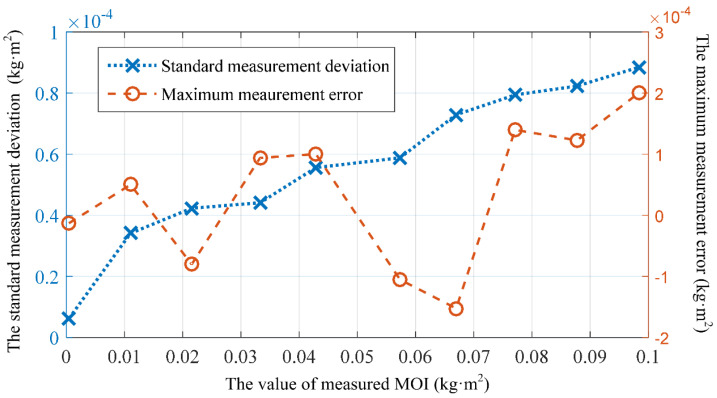
Standard measurement deviation and maximum measurement error for 10 measurements of different tested rotational inertia.

By stacking standard components or moving their position, the system can obtain different measured rotational inertia. In order to analyze measurement repeatability, correctness, and the effect of damping on the measurement results of the method and measurement system proposed in this paper, multiple sets of measurement experiments were conducted in 10 different load states with a range of rotational inertia from 0 to 100 × 10*^−^*^4^ kg·m^2^ and an inertia difference of about 0.01 kg·m^2^. Ten identical measurement experiments were conducted in each load state. Figure 12 shows the standard deviation and maximum measurement error of 10 measurements for each load state. As shown in the figure, the standard deviation of the measurement increases with increases in the measured rotational inertia value. Within the range of 0–100 × 10*^−^*^3^ kg·m^2^, the method proposed in this paper and the experimental system used can achieve an accurate measurement of the measured rotational inertia, the absolute value of the maximum measurement error is less than 2.00 × 10*^−^*^4^ kg·m^2^, and the standard deviation of the measurement is less than 0.90 × 10*^−^*^4^ kg·m^2^.

**Figure 13:**
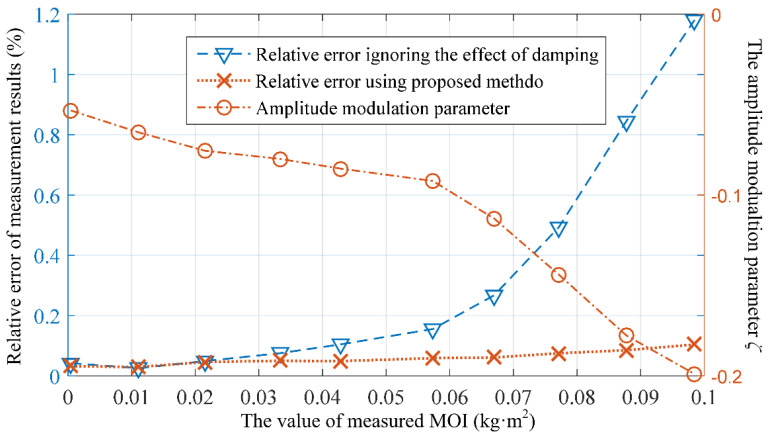
Relative error of the average value of the rotational inertia measurement results and the average value of the amplitude modulation parameter.

To observe the influence of torsion damping on the measurement of the rotational inertia, the relative measurement errors of the average of 10 measurements when neglecting damping and those considering damping using the method proposed in this paper (i.e., calculating the measured rotational inertia using Formula (8)) were both calculated. The results are shown in Figure 13, where the average values of the amplitude modulation parameter *ζ* for each tested load state are also given.

As shown in Figure 13, it can be observed that, within the experimental measurement range of 0–100 × 10^–3^ kg·m^2^, the influence of damping on the measurement results is insignificant when the measured load’s rotational inertia is relatively small. However, as the measured rotational inertia gradually increases, neglecting the effect of damping will result in increasingly larger measurement errors. The relative error of the average value of 10 measurements can exceed 1%, and the relative error of a single measurement is even greater. Therefore, the impact of damping cannot be ignored when using a low-cost measurement system (without air bearing). The proposed method and system in this paper significantly reduce the influence of damping on the measurements, and the relative error of the average value of 10 measurements is better than 0.1% within the experimental range. This measurement performance is comparable to that of a low-damping torsion pendulum measurement system equipped with an air bearing [21].

**Figure 14:**
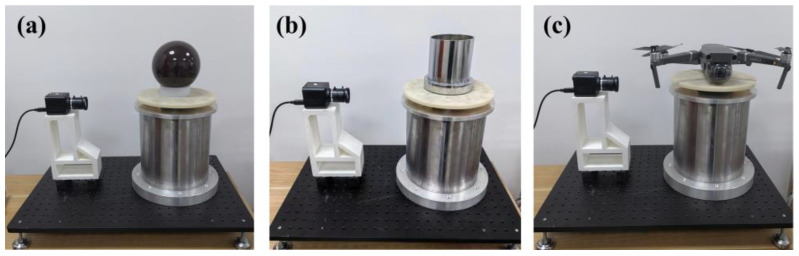
Experiments on measurement of rotational inertia of different identical parts: (**a**) standard round ball; (**b**) cylinder assemblies; (**c**) quadrotor UAV.

To further validate the effectiveness and applicability of the proposed method, rotational inertia measurement experiments were conducted on samples with different shapes and materials. As shown in Figure 14, the rotational inertia of a standard sphere, a sleeve assembly consisting of a metal cylindrical sleeve and a cylindrical base, and the Z-axis rotational inertia of a quadrotor UAV were measured. Table 3 presents the results of these rotational inertia measurements for the three types of loads. From Table 3, it can be observed that the method described in this paper enables stable rotational inertia measurements for samples with different shapes, and the standard deviation of the measurements remains at a low level.

**Figure 15:**
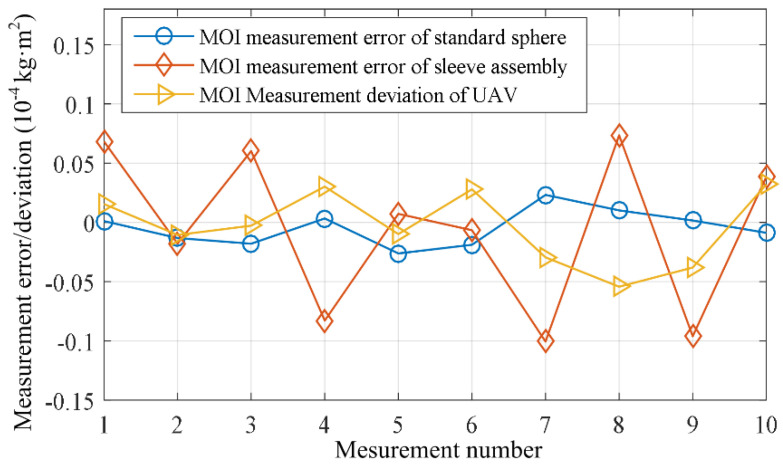
Inertia measurement error/deviation of three samples.

Among the three tested samples, the standard sphere and the sleeve assembly have regular shapes and uniform density. In this paper, their theoretical calculated values of rotational inertia are taken as the relative true values to calculate the measurement errors. The rotational inertia of the quadcopter around the Z-axis referenced from the specifications provided by the manufacturer is taken as the basis value to calculate the measurement deviations. The measurement errors or deviations for the 10 measurement experiments are calculated and shown in Figure 15. From the figure, it can be observed that the relative errors (or deviations) of the rotational inertia measurements for all three samples are better than 0.18% for a single measurement. These experiments demonstrate that the method and system described in this paper can adapt to the rotational inertia measurement requirements of various rigid bodies.

## 5. Conclusions

In contrast to previous studies, this paper innovatively applies machine vision technology to the measurement of the rotational inertia of rigid bodies. By accurately recording the angular displacement of torsional vibration using monocular vision, the identification of the torsional damping parameter (i.e., the amplitude modulation parameter) is achieved, thereby significantly reducing the impact of damping on rotational inertia measurements. An experimental system was constructed to validate and test the technical approach’s feasibility and measurement performance. The following conclusions were primarily obtained:

(1) A mathematical model for the measurement of rotational inertia using the torsion pendulum method was established. The solution to the torsional vibration differential equation under the assumption of linear damping was obtained, along with the relationship between the angular displacement, damping parameter, and rotational inertia measurement. A calculation model for rotational inertia considering damping was proposed.

(2) An experimental system based on monocular vision and a torsion pendulum platform was designed and developed. The rotational inertias of different rigid bodies were measured through experiments. The imaging system successfully captured the torsional vibration of the pendulum platform and obtained the torsional angular displacement time domain waveform. Based on the calculation model, the measured rotational inertia was determined, validating the feasibility of applying machine vision methods to rotational inertia measurement.

(3) Multiple measurement experiments were conducted on various types of rigid bodies, demonstrating the good measurement accuracy and repeatability of the proposed method. Within the range of 0–100 × 10*^−^*^3^ kg·m^2^, the standard deviation of the measurements was better than 0.90 × 10*^−^*^4^ kg·m^2^, and the absolute value of the measurement error was less than 2.00 × 10*^−^*^4^ kg·m^2^. The experimental results effectively demonstrate the effectiveness and applicability of the proposed method.

(4) The experimental results also revealed that neglecting damping would lead to measurement errors exceeding 1%. The proposed method successfully identifies system damping and corrects the rotational inertia calculation formula, resulting in a significant reduction in measurement errors. Moreover, the proposed system has a simple structure, low cost, and promising prospects for practical applications.

Furthermore, the experimental results also show that the absolute value of the damping factor increases with increases in the measured load, i.e., the amplitude attenuation of the torsion motion becomes more significant. This may be due to two reasons: (1) as the load increases, the internal damping of the elastic element during the torsion process increases (as manifested by heating and torsion fatigue of the elastic element), and thus the effect of damping is more significant than for smaller loads; (2) the mathematical model of damping used in this paper assumes linear damping, i.e., the damping torque is proportional to the angular velocity of the torsion motion. However, damping has various causes, and different forms of damping may occur under different conditions. Actual mathematical models of damping torque are more complex than the one used in this paper.

## Figures and Tables

**Table 1:** Main parameters of image system.

Equipment	Name of Parameter	Value of Parameter
Industrial Camera	Resolution	1920 px × 1200 px
	Frame Rate	60 FPS
	Pixel Size	4.8 μm
Camera Lens	Focal Length	16 mm
	Aperture	F1.4–16

**Table 2:** Results of calibration experiments.

Load State	Main Frequency of Torsion Pendulum (rad/s)	Amplitude Modulation Parameter	Relative True Value of Rotational Inertia
No-Load State	9.0017	−0.05197	--
Load State 1 (L = 0.05 m)	6.9930	−0.06017	7.4631 × 10^−3^
Load State 2 (L = 0.075 m)	5.8686	−0.06579	10.5966 × 10^−3^
Load State 3 (L = 0.10 m)	4.9352	−0.07123	14.9834 × 10^−3^
Calibration Results	*I*_0_*=* 4.5043 × 10^−3^ kg·m^2^; *k* = 0.3651 N·m		

**Table 3:** The moment of inertia measurement results of three samples (10^−4^ kg·m^2^).

No.	Standard Sphere	Sleeve Assembly	Quadrotor UAV
1	14.6872	46.0383	22.3655
2	14.6729	45.9521	22.3394
3	14.6681	46.0302	22.3472
4	14.6894	45.8871	22.3802
5	14.6599	45.9772	22.3401
6	14.6672	45.9634	22.3779
7	14.7093	45.8694	22.3205
8	14.6962	46.0435	22.2957
9	14.6877	45.8739	22.3119
10	14.6773	46.0084	22.3820
Standard deviation σ	0.0151	0.0679	0.0303

## Data Availability

The data presented in this study are available in the article.
